# Maladaptive Trained Immunity Drives Persistent IL-6 Production and Enhanced TLR Responsiveness in Monocyte-Derived Macrophages from People Living with HIV

**DOI:** 10.3390/microorganisms14020355

**Published:** 2026-02-03

**Authors:** Larisa Dubrovsky, Tatiana Pushkarsky, Beda Brichacek, Ashley Bastin, Afsoon Roberts, Jose Lucar, Maria Elena Ruiz, Oleksandr Semeniuk, Marc Siegel, Dmitri Sviridov, Michael I. Bukrinsky

**Affiliations:** 1 School of Medicine and Health Sciences, The George Washington University, Washington, DC 20037, USA; ld405@gwu.edu (L.D.); tpushk@gwu.edu (T.P.); bricbeda@gwu.edu (B.B.); ashleybastin@gwmail.gwu.edu (A.B.); aroberts@mfa.gwu.edu (A.R.); jlucar@mfa.gwu.edu (J.L.); mruiz@mfa.gwu.edu (M.E.R.); osemeniuk@gwu.edu (O.S.); msiegel@mfa.gwu.edu (M.S.); 2 Baker Heart and Diabetes Institute, Melbourne, VIC 3004, Australia; dmitri.sviridov@baker.edu.au; 3 Department of Biochemistry and Molecular Biology, Monash University, Clayton, VIC 3800, Australia

**Keywords:** HIV infection, trained immunity, inflammation, Nef, extracellular vesicles (EVs), PLWH, monocytes

## Abstract

Trained immunity (TRIM) enhances innate immune responses through epigenetic and metabolic reprogramming but may become maladaptive, contributing to chronic inflammation. In people living with HIV (PLWH), maladaptive TRIM has been proposed but remains insufficiently characterized. We examined inflammatory cytokine production in monocyte-derived macrophages (MDMs) obtained from PLWH and age-matched individuals without HIV infection. Baseline cytokine output and responses to stimulation of Toll-like receptors (TLR) were measured. We further examined whether TRIM influenced susceptibility to HIV infection in MDMs derived from monocytes exposed to extracellular vesicles carrying the HIV-1 Nef protein (Nef EVs). Baseline IL-6 production did not differ between unstimulated MDMs from PLWH and uninfected controls. Although sex-associated differences were initially observed, these effects were no longer significant after adjustment for infection duration. IL-6 responses following TLR2 and TLR7 stimulation, but not TLR4 stimulation, were significantly amplified in PLWH-derived MDMs, consistent with a trained phenotype. Similar trends were observed in sex-stratified analyses but did not reach statistical significance. The magnitude of unstimulated IL-6 production positively correlated with duration of HIV infection, suggesting cumulative TRIM imprinting over time. Despite heightened inflammatory responsiveness, TRIM did not reduce susceptibility to HIV infection in Nef EV-exposed MDMs, indicating functional maladaptation rather than protective priming. These findings provide evidence of maladaptive TRIM in PLWH, characterized by preserved basal cytokine output but exaggerated inflammatory responses to innate immune stimulation without antiviral benefit. The association with infection duration supports progressive innate immune reprogramming as a contributor to HIV-associated inflammation. No statistically significant differences in trained immune responses were observed between male and female PLWH after accounting for duration of infection. Further studies are needed to define the mechanisms underlying this maladaptation and its clinical consequences.

## 1. Introduction

Trained immunity (TRIM), a form of innate immune memory, is characterized by the enhanced responsiveness of innate immune cells to subsequent infections following an initial stimulus. This phenomenon is mediated by epigenetic, metabolic, and functional reprogramming of innate immune cells, such as monocytes and natural killer cells, enabling them to mount stronger responses to secondary challenges [1]. While trained immunity is widely acknowledged to play a beneficial role in host defense, emerging evidence indicates that, in certain contexts, it may also contribute to adverse outcomes, including chronic inflammation and inflammation-associated comorbidities [2,3]. This phenomenon has been termed maladaptive trained immunity, referring to a persistent state of memory imprinted in innate immune cells that, instead of eliciting protective responses, results in detrimental effects such as sustained inflammation and immune dysregulation.

This duality is particularly relevant in the context of chronic viral infections, such as HIV, where maladaptive TRIM may promote persistent immune activation and low-grade inflammation [4]. These features are characteristic to antiretroviral therapy (ART)-suppressed HIV infection [5] and contribute to an increased risk of HIV-associated comorbidities. Chronic immune activation can also lead to immune exhaustion and increased susceptibility to secondary infections [6] or autoimmune-like conditions [7], further exacerbating the burden of HIV-related comorbidities.

Analysis of innate immune cells in PLWH supports the acquisition of trained immunity. A study by Bowman et al. [8] demonstrated enhanced inflammatory cytokine production by monocyte-derived macrophages (MDMs) obtained from PLWH compared to uninfected controls. This increase was associated with the activation of genes regulating inflammatory proteins and lipid metabolism, both of which are key hallmarks of trained immunity [9]. These findings were further corroborated by van der Heijden et al. [10], who reported that monocytes from PLWH undergo transcriptional reprogramming characteristic of trained immunity. Additionally, Jost et al. [11] identified natural killer (NK) cells with memory-like features in PLWH, further supporting the presence of trained immunity in this population.

HIV infection, even when effectively suppressed by ART, is associated with the presence of circulating extracellular vesicles (EVs) carrying the viral protein Nef [12,13,14]. Notably, Nef-positive EVs were detected in 50% of samples from ART-treated individuals with undetectable viral loads [13]. These EVs have been implicated in immune modulation, altering the function of bystander immune cells and promoting pro-inflammatory states [15]. Specifically, Nef EVs have been shown to reprogram monocytes and macrophages, influencing lipid metabolism and cytokine production in a manner consistent with the induction of trained immunity [16]. Nef EV-induced reprogramming of myeloid cells is likely a significant contributor to TRIM in PLWH [4].

This study was undertaken to characterize the functional changes in monocytes from PLWH. By comparing ex vivo production of cytokines by monocytes from ART-treated PLWH to those from uninfected controls, we sought to characterize trained immunity in PLWH and define its correlates. Furthermore, we investigated sex-specific differences in trained immunity, a previously unexplored aspect in this context. Additionally, we examined the susceptibility of monocytes trained by Nef EVs to HIV infection. Collectively, this study provides novel insights into the mechanisms and implications of trained immunity in the context of HIV infection.

## 2. Materials and Methods

*Study Participants:* We enrolled 40 ART-treated PLWH and 36 age- and sex-matched controls without HIV infection (Table 1). All PLWH were receiving antiretroviral therapy, with the following class distribution: nucleoside reverse transcriptase inhibitors (NRTIs; 97%), nucleotide reverse transcriptase inhibitors (NtRTIs; 10%), non-nucleoside reverse transcriptase inhibitors (NNRTIs; 37%), protease inhibitors (PIs; 3%), and integrase strand transfer inhibitors (INSTIs; 90%). All the participants signed the HIV Specimen Bank Informed Consent consenting to the use of their clinical information. The study was approved by GWU IRB, approval #021848. All PLWH had undetectable viral loads (<20 copies/mL) and were on stable ART for at least 6 months.

*MDMs, viruses and infection:* Buffy coats from healthy donors were obtained from the Gulf Coast Blood Center. Peripheral blood mononuclear cells (PBMCs) were isolated from whole blood using density gradient centrifugation, monocytes were purified by adherence to plastic and differentiated into MDM as described [16]. Briefly, PBMCs were isolated by density centrifugation using Ficoll-Paque PLUS density gradient media (Cytiva, Marlborough, MA, USA, cat. # 17144002) and, after washing 3 times with PBS (Gibco, Waltham, MA, USA, cat. # 10010023), cells (9 × 10^6^/mL) were plated in Primaria plates in RPMI 1640 medium (Gibco, cat. # 11875093) supplemented with 1% Pen/Strep (Corning, Corning, NY, USA, cat. # 30-001-CI), 10 mM L-Glutamine (Corning, cat. # 25-005-CI) and 10 mM sodium pyruvate (Corning, cat. # 25-000-CI) and incubated in a humidified 37 °C incubator with 5% CO_2_ for 2 h to adhere to plastic. Non-adhered cells were removed by washing 3-times with warm DPBS (Gibco, with Mg^2+^ and Ca^2+^, cat. # 140401330). Adhered cells were washed with warm PBS and exposed to a fresh complete medium (supplemented with 20 ng/mL M-CSF (Sigma, St. Louis, MO, USA, cat. #SRP3110-10UG) for the next 6 days. Information regarding donor sex or age was not provided. MDMs were infected with laboratory-adapted HIV-1 subtype B strains ADA [17] and AD8 [18], or with the transmitted/founder (T/F) subtype C viruses Z331M and Z4473M [19] (NIH HIV Reagent Program), using an inoculum of 20 ng p24 per 10^6^ cells as described [20,21]. The ADA strain was propagated in PBMCs from uninfected donors, whereas AD8 and the T/F viruses were amplified by transfection of HEK293T cells with their respective molecular clones.

**Figure A1:**
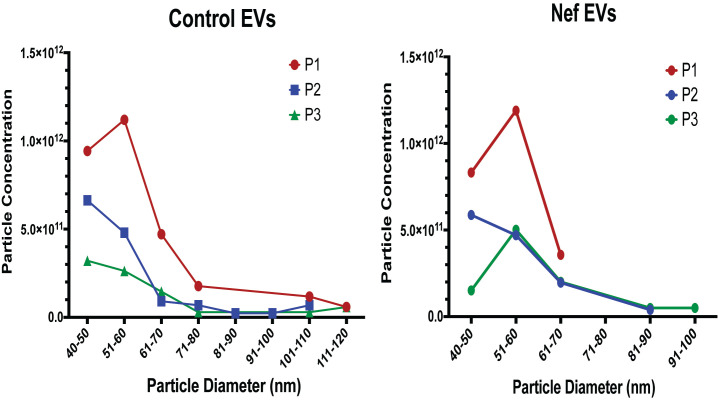
Analysis of EVs using the Izon Exoid instrument.

*EV preparation and analysis:* Nef EVs and control EVs were prepared from transfected HEK293T cells as described [22]. These EV preparations were generated using a differential centrifugation protocol previously validated by our group to yield vesicles positive for canonical EV markers (including Alix, CD63 and CD81) and devoid of detectable cellular contaminants [22]. EVs were quantified using the Izon Exoid instrument(Izon Science, Christchurch, New Zealand), which employs Tunable Resistive Pulse Sensing (TRPS) to measure particle diameter and concentration. Before sample analysis, a calibration standard was loaded to optimize nanopore stretch, pressure, and voltage parameters, ensuring accurate sizing within the target range. As each EV traverses the nanopore, the Exoid software (version 1.5) records a transient current drop (“blockade”), with blockade magnitude and frequency corresponding to particle size and concentration, respectively. During TRPS acquisition, measurements are collected at three pressure settings (P1, P2, and P3), allowing the software to account for pressure-dependent effects on particle flow and thereby refine the final concentration estimate (Appendix A Figure A1). After data acquisition, files are exported into the Izon Exoid Data Suite, where measurements are processed, calibrated against the standard, and converted into final size and concentration outputs.

*Treatment of monocytes with EVs:* Monocytes from healthy donors were treated with 5 × 10^9^ particles/mL of Nef-containing EVs or control EVs for 48 h, washed, and then differentiated into MDMs for 5 days. To assess EV-induced functional reprogramming, the resulting MDMs were stimulated with TLR agonists and IL-6 levels in the supernatants were quantified by ELISA. To evaluate the impact of EV exposure on susceptibility to HIV infection, MDMs were infected with HIV-1, and viral replication kinetics were monitored by measuring p24 levels in the culture supernatant by ELISA.

*Cytokine Production Assays:* MDMs from PLWH and controls without HIV infection were treated for 24 h with TLR agonists: lipopolysaccharide (LPS, 2 ng/mL) for TLR4, R-848 (500 ng/mL) for TLR7/8, and Pam3CSK4 (10 ng/mL) for TLR2. Untreated MDMs served as baseline controls. Supernatants were collected after 24 h, and IL-6 and TNFα levels were quantified using enzyme-linked immunosorbent assays (ELISAs) following the manufacturer’s instructions (Promega, Madison, WI, USA).

*Statistical Analysis:* Data were analyzed using GraphPad Prism 10. Comparisons between groups were performed using ANOVA, with *p* < 0.05 considered statistically significant. Data are presented as mean ± SEM.

## 3. Results

The control group recruited for this study was age-matched to the PLWH group (Table 1). Plasma viral load was below the limit of detection (20 copies/mL) in all PLWH. Within male and female subgroups, no significant age differences were observed. However, a significant racial disparity was noted between the control and PLWH groups: while white individuals made up 50% of the control group, they were a clear minority in the PLWH group.

We first measured IL-6 and TNFα levels produced by macrophages differentiated ex vivo from monocytes of PLWH and controls without HIV infection, without any additional stimulation. Although no external stimulants were added, low-level basal cytokine production was detected, consistent with a low level of metabolic activity observed in unstimulated macrophage cultures [23]; this activity was comparable between PLWH and control groups and therefore did not affect group comparisons.

To evaluate the influence of race and sex, we conducted a multiple linear regression analysis examining the association between HIV status and log-transformed IL-6 and TNFα levels, adjusting for sex and race. For IL-6 (Table 2), the overall model was statistically significant (F(6,59) = 2.758, *p* = 0.0197), explaining 21.91% of the variance (R^2^ = 0.2191).

While HIV status alone was not significantly associated with IL-6 levels (β = −0.0157, *p* = 0.9505), sex was a significant predictor, with females exhibiting higher IL-6 levels than males (β = 0.6557, *p* = 0.0058). Additionally, a significant interaction between HIV status and sex (β = −0.8769, *p* = 0.0146) suggested that the effect of HIV on IL-6 levels differs between males and females. Race did not have a significant effect (β = 0.0144, *p* = 0.9704). For TNFα levels, no significant associations were found, and none of the independent variables, including HIV status, significantly predicted TNFα levels (Appendix A Table A1). Based on these findings, our subsequent analyses focused on IL-6.

**Figure 1:**
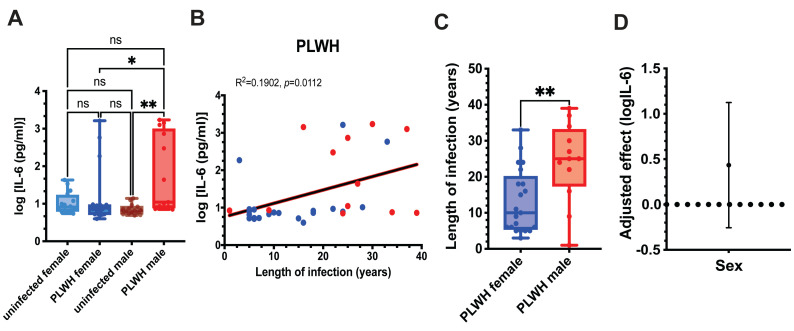
Sex-Specific Differences in Cytokine Responses of MDMs from PLWH. Monocytes from PLWH and uninfected controls were differentiated into MDMs ex vivo. (**A**)—IL-6 levels were measured in the supernatants of unstimulated MDMs derived from monocytes of PLWH and uninfected controls, with results stratified by sex. Data are shown as box-and-whisker plots, where boxes represent the interquartile range (IQR), the horizontal line indicates the median, and whiskers denote the minimum and maximum values. Statistical significance was determined using the Kruskal–Wallis non-parametric ANOVA test, with *p*-values indicated (* *p* = 0.0284; ** *p* = 0.0025). ns indicates non-significant difference. (**B**)—Linear regression analysis showing the correlation between the logarithm of IL-6 levels produced by unstimulated MDMs from PLWH and the duration of infection. Red dots represent males, and blue dots represent females. (**C**)—Comparison of the length of infection between male and female PLWH in our cohort by Mann–Whitney test. ** *p* = 0.0062. (**D**)—Linear regression analysis estimating the effect of sex on log-transformed IL-6 production, adjusted for duration of HIV infection. The plotted point represents the regression coefficient comparing males to females, with error bars indicating the 95% confidence interval. The horizontal zero line denotes the null value, where no sex difference in IL-6 levels would be observed following adjustment.

Given the interaction between HIV status and sex, we next compared IL-6 levels produced by unstimulated MDMs from PLWH and uninfected controls within sex-stratified subgroups (Figure 1A). Male and female subgroups of PLWH did not differ significantly in CD4+ T cell counts, indicating comparable immune status (Table 1) and excluding this factor as a potential confounder. Male PLWH exhibited significantly higher IL-6 levels than both uninfected male and female PLWH, whereas IL-6 production in female PLWH was comparable to that in uninfected female controls (Figure 1A).

We next examined whether unstimulated IL-6 production was associated with duration of HIV infection. Across all PLWH, IL-6 levels showed a significant positive correlation with years since HIV diagnosis (Figure 1B), consistent with cumulative inflammatory imprinting. To determine whether infection duration could account for the sex differences observed in Figure 1A, we compared the length of HIV infection between male and female PLWH. Men in our cohort had been infected significantly longer than women (Figure 1C), raising the possibility that infection duration contributes to the elevated IL-6 production observed in males.

To formally assess whether sex remained an independent predictor of IL-6 production after accounting for infection duration, we performed linear regression analysis adjusting for years since diagnosis. Inclusion of infection duration attenuated the regression coefficient for sex, and the 95% confidence interval crossed zero, indicating that the sex difference was no longer statistically significant (Figure 1D).

Together, these results indicate that IL-6 production by unstimulated MDMs does not differ significantly between PLWH and uninfected controls when analyzed without sex stratification. However, sex-stratified analyses revealed elevated IL-6 production in male PLWH, which correlated with longer infection duration. After adjustment for infection duration, sex was no longer an independent predictor of IL-6 levels, supporting infection duration as a key determinant of IL-6 production in this cohort.

**Figure 2:**
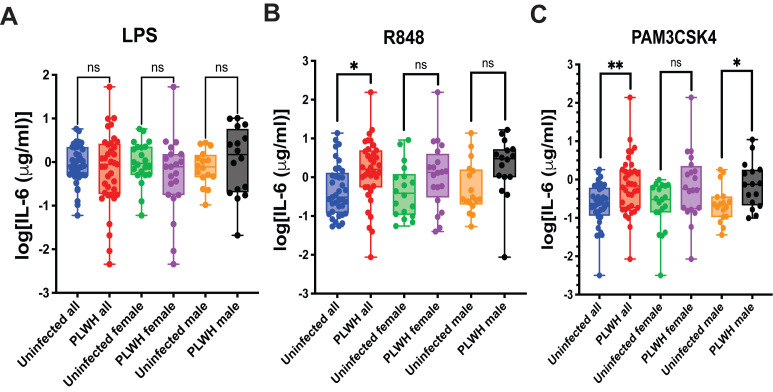
Responses of MDMs from PLWH to TLR Agonists. Monocytes from PLWH and uninfected controls were differentiated into MDMs ex vivo and stimulated with the indicated TLR ligands. IL-6 levels were measured by ELISA. (**A**)—IL-6 production in response to LPS stimulation, analyzed for all samples and stratified by sex. (**B**)—IL-6 production in response to R848 stimulation, analyzed for all samples and stratified by sex (* *p* = 0.0200). (**C**)—IL-6 production in response to Pam3CSK4 stimulation, analyzed for all samples and stratified by sex (* *p* = 0.0464; ** *p* = 0.0047; ns—non-significant). All comparisons were performed using ordinary one-way ANOVA with Sidak’s post hoc correction for multiple comparisons.

A classical test for the trained immunity phenotype is an increased response of trained myeloid cells to stimulation by TLR agonists [24]. To evaluate this, we MDMs differentiated from monocytes obtained from PLWH or uninfected controls to stimulation by LPS (TLR4 agonist), R848 (TLR7/8 agonist), or Pam3CSK4 (TLR2 agonist) and measured IL-6 production. This analysis was performed both with and without separating the samples by sex. The results are presented in Figure 2.

Stimulation of MDMs with LPS produced comparable IL-6 responses in PLWH and uninfected controls, with no significant differences observed in the overall cohort or within sex-stratified groups (Figure 2A). In contrast, R848 stimulation resulted in significantly higher IL-6 production in PLWH compared with uninfected individuals in the combined cohort (*p* = 0.0453), while the female and male subgroup comparisons showed only non-significant trends toward increased cytokine levels in PLWH (Figure 2B). Following Pam3CSK4 stimulation, IL-6 production was significantly elevated in PLWH in the overall cohort and remained significant among women, whereas the difference in men did not reach statistical significance and exhibited only a trend (Figure 2C). Overall, these findings indicate that MDMs from PLWH show heightened responsiveness to selected TLR ligands, a characteristic feature of trained immunity.

These findings are consistent with those reported by Bowman et al. [8] and suggest that monocytes circulating in the peripheral blood of PLWH are intrinsically predisposed to produce elevated levels of inflammatory cytokines upon differentiation and stimulation. This pattern is indicative of a trained immunity-like state, in which myeloid cells are durably reprogrammed to mount exaggerated pro-inflammatory responses. Importantly, such reprogramming is best interpreted as a maladaptive form of TRIM, as it promotes persistent inflammation rather than an acute, protective inflammatory response to activating stimuli.

While adaptive TRIM is a protective mechanism that can restrict viral replication [25], the impact of maladaptive TRIM on the susceptibility of myeloid cells to HIV infection remains poorly understood. Because only limited volumes of blood could be obtained from study participants, we were unable to directly assess HIV susceptibility in MDMs from PLWH in this cohort. To address this experimental limitation, we used Nef-containing extracellular vesicles (Nef EVs) as a mechanistic surrogate to model HIV-associated inflammatory training in myeloid cells. Nef EVs were selected for this purpose because we previously demonstrated that they reproducibly induce durable inflammatory reprogramming consistent with maladaptive TRIM [16]. We then compared HIV susceptibility in MDMs differentiated from Nef EV–treated monocytes and those treated with control EVs. Importantly, Nef EV–treated MDMs represent a reductionist experimental model used to probe the functional consequences of HIV-associated inflammatory training and are not intended to fully recapitulate the complex, long-term trained immunity phenotype observed in PLWH.

**Figure 3:**
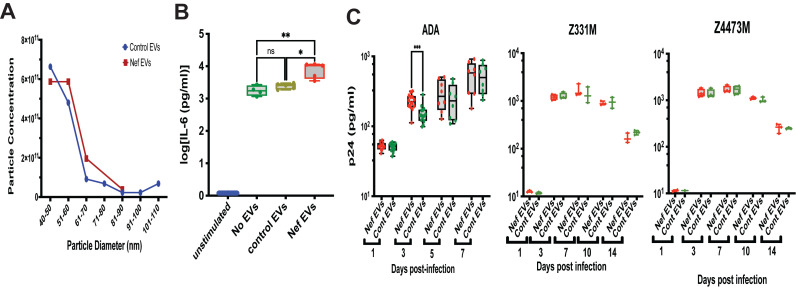
Effect of Nef EVs on MDM susceptibility to HIV infection. Monocytes from an uninfected donor were treated with Nef EVs or control EVs, differentiated into macrophages, and infected with HIV-1. Viral replication was monitored by measuring p24 levels in culture supernatants by ELISA. (**A**)—Characteristics of EV preparations as determined by Izon Exoid analysis. (**B**)—Monocytes were seeded in 4–5 wells, treated with EVs, differentiated into MDMs, and stimulated with LPS. IL-6 levels in the supernatant were quantified by ELISA. Data are presented as box-and-whisker plots, where boxes represent the interquartile range (IQR), the horizontal line denotes the median, and whiskers extend from the minimum to the maximum values. Statistical analysis was performed using Brown–Forsythe and Welch ANOVA with Dunnett’s correction for multiple comparisons. * *p* = 0.0053, ** *p* = 0.0003, ns—non-significant. *n* = 4 (no EVs) and *n* = 5 (Nef EVs and control EVs). (**C**)—Monocytes were treated as in panel B and infected with the indicated HIV-1 strains. Viral replication was assessed by p24 ELISA. Data are shown as box-and-whisker plots (IQR with median; whiskers from min to max) and analyzed by unpaired *t*-test. *** *p* = 0.0006. *n* = 8–14 (ADA) and *n* = 3–4 (Z331M and Z4473M).

The characteristics of the EV preparations are shown in Figure 3A; no significant differences in particle size or concentration were observed between control and Nef EVs. To confirm functional competence, we performed a previously established assay [16] assessing the ability of EVs to induce pro-inflammatory memory in monocyte-derived macrophages. As shown in Figure 3B, monocytes exposed to Nef EVs exhibited an enhanced IL-6 response to LPS stimulation, confirming expected biological activity of Nef EVs to induce pro-inflammatory memory in MDMs [16].

**Figure A2:**
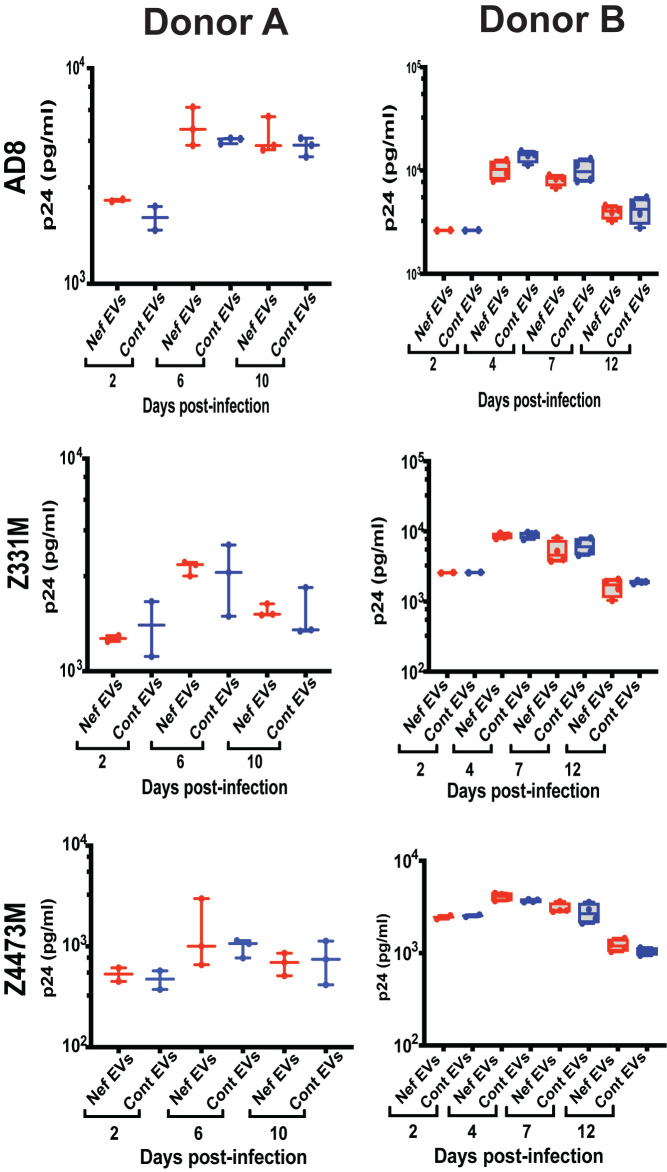
Analysis of HIV-1 replication in MDMs from two donors.

Replication of three HIV-1 strains—the laboratory-adapted ADA strain [26] and the transmitter-founder (T/F) subtype C strains Z331M [27] and Z4473M [19]—was similar in MDMs derived from Nef EV–treated and control EV–treated monocytes (Figure 3C). A modest early increase in ADA replication at 3 days post-infection in Nef EV–conditioned MDMs did not persist at later time points and was not reproducible across different donors (Appendix A Figure A2).

Collectively, these results demonstrate that Nef EV–induced trained immunity does not confer protection against HIV infection, consistent with its characterization as maladaptive TRIM.

## 4. Discussion

Trained immunity (TRIM) has evolved as a protective mechanism that increases responses to infection. However, several recent reports demonstrated that TRIM can lead to persistent inflammation and inflammation-associated co-morbidities [2,28,29]. Our analysis demonstrated that IL-6 production by unstimulated MDMs derived from PLWH monocytes directly correlated with the duration of HIV infection, suggesting that longer infection is associated with a progressively heightened basal inflammatory state in monocytes. Nevertheless, no overall difference in IL-6 production between PLWH and uninfected controls was observed. Sex-stratified analyses revealed that elevated IL-6 levels were confined to male PLWH in our cohort, who had significantly longer infection duration than females. The significant association between IL-6 production and years since diagnosis, together with the loss of statistical significance for the sex effect after adjustment for infection duration, indicates that infection duration, rather than biological sex per se, is the primary driver of enhanced inflammatory responses. These findings support a model in which inflammatory activation intensifies cumulatively over the course of HIV infection, potentially reflecting progressive inflammatory imprinting. The absence of a global difference between PLWH and controls likely reflects the predominance of relatively recently infected individuals in our cohort, particularly among women, whose IL-6 levels remained comparable to uninfected controls. Overall, these results highlight the importance of accounting for infection duration when evaluating inflammatory profiles in PLWH and suggest that long-term HIV infection may lead to persistent inflammatory activation even under virological suppression.

Our analyses focused on IL-6 because TNF-α did not show significant associations with HIV status, sex, race, or infection duration. This divergence is biologically informative rather than a limitation. IL-6 and TNF-α play distinct roles in inflammatory responses, and their differential regulation is consistent with the concept of maladaptive trained immunity in chronic HIV infection.

TNF-α is a prototypical early-response cytokine that mediates acute inflammation, pathogen clearance, and cytotoxic effector functions [30]. Its production is typically rapid, transient, and tightly regulated, reflecting its potential for tissue damage if sustained. In contrast, IL-6 is a central mediator of chronic, low-grade inflammation, integrating metabolic, epigenetic, and inflammatory signals and promoting long-term immune reprogramming [31,32]. IL-6 is also a key driver of systemic inflammatory states associated with aging, cardiovascular disease, and other HIV-associated comorbidities [33,34]. Thus, the selective enhancement of IL-6, but not TNF-α, observed in our study is consistent with a maladaptive form of trained immunity characterized by chronic inflammatory imprinting rather than heightened acute responsiveness.

Importantly, IL-6 production correlated with duration of HIV infection, further supporting its role as a cumulative marker of long-term inflammatory training. The absence of a similar relationship for TNF-α suggests that HIV-associated TRIM does not broadly amplify all inflammatory pathways, but instead selectively reinforces those linked to persistent inflammation and immunometabolic dysregulation. This selective targeting may explain why maladaptive TRIM contributes to chronic inflammation and comorbidity risk in PLWH without conferring enhanced antiviral or antimicrobial protection.

Notably, since MDM differentiation was conducted ex vivo without factors promoting M1 polarization (the M-CSF added to the medium supports cell survival and promotes M2 differentiation [35]), the observed inflammatory phenotype reflects an inherent predisposition of monocytes of PLWH toward inflammatory responses. This suggests that these monocytes may be “trained” to respond in a pro-inflammatory manner. IL-6 production is characteristic of chronic inflammation [36], whereas TNFα is more associated with acute inflammatory events [37], suggesting that monocytes are reprogrammed for sustained inflammatory responses, a hallmark of maladaptive TRIM. This finding aligns with that reported by van der Heijden et al. [10], whose cohort was predominantly male, and suggests that monocytes circulating in the peripheral blood of male PLWH are intrinsically predisposed to produce inflammatory cytokines, in particular IL-6. This state may be a result of continuous production of pro-inflammatory monocytes by the bone marrow, due to inflammatory memory in myeloid progenitor cells, or reflect an incomplete return to the resting state of activated monocytes in the periphery. Our analyses did not detect increased levels of CD14, CD143 or CRP in the blood of PLWH relative to uninfected controls , arguing for the trained immunity explanation and against the continuous stimulation of peripheral monocytes in our cohort.

We observed that MDMs from PLWH, when stimulated with the TLR7/8 agonist R848 or the TLR2 agonist Pam3CSK4, produced higher levels of IL-6 compared to MDMs from controls without HIV infection. When stratified by sex, the differences lost statistical significance, but the trend was observed. This is likely due to limited number of participants in our cohort. TLR7/8 recognize single-stranded RNA from viruses such as HIV, influenza, and SARS-CoV-2, triggering the production of Type I interferons (IFN-α and IFN-β) to enhance antiviral immunity [38]. In contrast, TLR2 detects a broad range of microbial molecules, including those from bacteria, fungi, and some viruses. Upon activation, TLR2 signals through the MyD88 adaptor protein, leading to NF-κB and MAPK pathway activation, which drives inflammatory responses [39]. Interestingly, we did not observe differences in LPS-stimulated responses between PLWH and uninfected cohorts, whereas van der Heijden et al. reported a significant increase in LPS-induced cytokine production by MDMs from PLWH [10]. The cohort analyzed by van der Heijden et al. [10] was predominantly male, and in our study, the male PLWH subgroup also exhibited a trend toward increased LPS-induced IL-6 production. However, this difference did not reach statistical significance, possibly due to the limited number of male participants. Differences in ART regimen composition could, in principle, also influence innate immune activation and trained immunity phenotypes. However, both our and van der Heijden et al. [10] cohorts were highly similar with respect to backbone therapy, with nearly universal use of nucleoside reverse transcriptase inhibitors (NRTIs) (96–97%). While some differences were observed in the use of individual ART classes, most notably a higher prevalence of integrase strand transfer inhibitors (INSTIs) in our cohort (90% vs. 67%) and lower use of protease inhibitors (PIs) (3% vs. 15%), these differences do not provide a clear mechanistic explanation for the divergent LPS responses.

Importantly, no ART class has been consistently linked to selective amplification or suppression of TLR4-mediated cytokine responses in ART-suppressed PLWH, and INSTI-based regimens are generally associated with reduced, rather than heightened, systemic inflammation. Moreover, trained immunity reflects cumulative inflammatory imprinting that integrates exposures across the course of infection, including periods preceding the current ART regimen. As such, cross-sectional comparisons based on current ART class distribution are unlikely to capture the determinants of LPS responsiveness.

These observations suggest that the discrepancy between our and van der Heijden et al. [10] studies more likely reflects cohort-specific biological factors, such as infection duration, comorbidity burden, microbial translocation, or host metabolic state, rather than differences in ART regimen composition alone. Definitive assessment of ART-specific effects on trained immunity will require larger, prospectively designed studies with sufficient power to stratify by regimen history and relevant clinical covariates.

Our study demonstrated that MDMs trained by Nef EVs did not develop enhanced resistance to HIV infection. This is in contrast to classical adaptive forms of trained immunity, in which prior stimulation strengthens antimicrobial defenses and reduces pathogen replication [25]. Despite exhibiting a heightened inflammatory phenotype following Nef EV exposure, the MDMs showed no reduction in replication of multiple HIV-1 strains, indicating that the inflammatory reprogramming induced by Nef does not translate into improved antiviral control. Instead, the transient increase in viral replication observed at early time points in some experiments suggests that Nef-induced inflammatory conditioning may even create a more permissive environment for infection. These findings underscore the distinction between protective TRIM and the maladaptive form induced by HIV Nef, in which inflammatory activation is amplified but does not confer functional resistance to subsequent HIV challenge. Of note, Nef EV exposure models a discrete component of HIV-associated immune training rather than its entirety. Despite this limitation, the Nef EV model remains a valid and informative experimental system because it isolates a biologically relevant HIV-associated stimulus and allows direct testing of the functional consequences of inflammatory training.

The potential contribution of race to trained immunity is an important and complex question. Because race was unevenly distributed between groups, our study was not powered to detect modest race-associated effects, and race-related analyses should therefore be interpreted cautiously. Within these constraints, our regression analysis did not identify race as an independent predictor of IL-6 production, arguing against race itself acting as a primary driver of trained immunity in this cohort. If racial identity alone were sufficient to determine inflammatory programming, a main effect of race would be expected; however, no such effect was observed.

Instead, these findings suggest that any influence of socioeconomic or psychosocial stressors on trained immunity is likely indirect and heterogeneous, and not adequately captured by race as a categorical variable. Social determinants of health vary widely within racial groups and may shape innate immune programming through cumulative exposures rather than through race per se. As a result, race represents an imprecise surrogate for the specific stressors that contribute to inflammatory imprinting, particularly in modestly sized cohorts.

Consistent with this interpretation, duration of HIV infection emerged as a significant correlate of IL-6 production, indicating that prolonged biological exposure to HIV-associated inflammatory stimuli is a dominant and measurable driver of trained immunity in this study. Any additional effects of socioeconomic stress are therefore likely secondary to, or integrated with, infection-related inflammatory imprinting rather than operating as independent predictors detectable in our model.

An important unresolved question is when maladaptive trained immunity arises: during untreated HIV infection, at the time of antiretroviral therapy (ART) initiation, or during long-term viral suppression. While the design of our study does not allow us to pinpoint a single stage at which maladaptive TRIM is established, several observations support a model in which it is progressively acquired over the course of HIV infection and persists during long-term ART-mediated viral suppression. First, all participants in our cohort were virologically suppressed, yet IL-6 production by monocyte-derived macrophages correlated positively with duration of HIV infection, indicating cumulative inflammatory imprinting despite effective viral control. Moreover, the absence of a global difference in basal IL-6 production between PLWH and uninfected controls argues against a transient inflammatory state confined to early infection or ART initiation, which would be expected to affect all PLWH similarly. Instead, the dependence of IL-6 production on infection duration supports a model of progressive, cumulative inflammatory imprinting. Finally, the persistence of exaggerated cytokine responses to selected Toll-like receptor agonists after ex vivo differentiation of monocytes from ART-suppressed individuals indicates a stable, cell-intrinsic reprogramming that is maintained in the absence of ongoing infection. This pattern is characteristic of durable, memory-like innate immune training rather than transient acute activation. Together, these findings support the concept that maladaptive TRIM reflects long-term integration of inflammatory exposures across the course of HIV infection and is maintained during virologic suppression.

In conclusion, our findings provide evidence for the presence of trained immunity in PLWH and characterize its maladaptive nature, marked by sustained inflammatory responsiveness without a corresponding protective effect against HIV infection. While our data are consistent with maladaptive trained immunity, we acknowledge that additional mechanisms of chronic immune activation, such as persistent low-level antigenic stimulation, microbial translocation, or ongoing presence of circulating Nef EVs in some PLWH, may also contribute to sustained inflammation in this population. The persistence of heightened cytokine responses following ex vivo differentiation, together with their association with duration of HIV infection, supports durable innate immune reprogramming, although these observations do not exclude the coexistence of other inflammatory drivers.

Future studies will be required to disentangle the relative contributions of trained immunity and other forms of chronic immune activation. Longitudinal analyses spanning untreated infection, antiretroviral therapy initiation, and long-term viral suppression will be critical for defining the temporal dynamics of maladaptive immune programming. In parallel, mechanistic studies directly interrogating epigenetic, metabolic, and transcriptional hallmarks of trained immunity in myeloid cells from PLWH will help establish causal links between inflammatory imprinting and functional outcomes. Finally, pre-clinical and clinical studies testing interventions that target trained immunity-associated pathways may determine whether modulation of innate immune memory can reduce chronic inflammation and mitigate HIV-associated comorbidities. Together, these findings underscore the need for targeted therapeutic strategies aimed at restoring immune homeostasis and limiting the long-term consequences of chronic inflammation in PLWH.

## Figures and Tables

**Table 1:** Study participants.

Group	*n*	Age (Years ± SD)	*p* Value (Age: PLWH vs. Control)	Race (Black/White)	CD4+ T Cells (Cells/mL) ^1^	VL (Copies/mL) ^1^
PLWH (total)	40	57.6 ± 5.6	ns ^3^	34/6	862.2 ± 387.4	<20
PLWH (male)	16	58.1 ± 6.1	ns	13/3	781.3 ± 304.8	<20
PLWH (female)	24	57.2 ± 5.3	ns	21/3	918.5 ± 433.4	<20
Controls (total)	36	58.7 ± 5.9	ns	17/19	ND ^2^	
Controls (male)	20	59.4 ± 5.9	ns	10/10	ND	
Controls (female)	16	58.1 ± 5.9	ns	8/8	ND	

^1^ Data were collected at the time of blood sampling for the study. ^2^ ND—not done. ^3 ^ns—not significant.

**Table 2:** Multiple Linear Regression Analysis Predicting logIL-6 Levels.

Predictor	B (Estimate)	t	*p*	95% CI
HIV Status (PLWH vs. Uninfected)	−0.0157	0.062	0.950	[−0.345, 0.313]
**Gender (Female vs. Male)**	**0.6557**	**2.864**	**0.006**	**[0.198, 1.113]**
Race (Black vs. White)	0.0144	0.037	0.970	[−0.374, 0.403]
**HIV Status × Gender**	**−0.8769**	**2.516**	**0.015**	**[−1.580, −0.174]**
HIV Status × Race	−0.1296	0.316	0.753	[−0.852, 0.593]
Gender × Race	0.0893	0.235	0.815	[−0.628, 0.807]

Note: B—Unstandardized coefficient; CI—Confidence interval (95%); t—t-statistic calculated as t = coefficient estimate/standard error of the estimate (tests whether the estimated effect of a predictor is significantly different from zero). Significant predictors are bolded.

## Data Availability

The original contributions presented in this study are included in the article. Further inquiries can be directed to the corresponding author.

## Abbreviations

The following abbreviations are used in this manuscript:

HIV	Human Immunodeficiency Virus
PLWH	People living with HIV
PBMC	Peripheral blood mononuclear cells
MDM	Monocyte-derived macrophages
EV	Extracellular vesicle
TRIM	Trained immunity
NK cells	Natural killer cells
ART	Anti-retroviral therapy
TLR	Toll-like receptor
LPS	Lipopolysaccharide

## Appendix A

Graphs show the size and concentration profiles of control EVs and Nef EVs measured at three pressure settings (P1, P2, and P3) on the Exoid instrument.

MDMs were generated from PBMCs of two uninfected donors and infected in vitro with the indicated HIV-1 strains. Graphs show box-and-whisker plots of p24 levels in culture supernatants at the indicated time points post-infection, with whiskers extending from the minimum to the maximum values.

MDMs were derived from monocytes treated with Nef EVs (red) or Control EVs (blue). p24 levels were measured by ELISA at indicated timepoints. Data represent mean ± SEM from 2–4 independent measurements.

**Table A1:** Multiple Linear Regression Analysis of logTNFα.

Predictor	B	*t*	*p*
Intercept	1.125	5.440	<0.0001
HIV Status (0 = Uninfected, 1 = HIV+)	0.4146	1.155	0.2527
Gender (0 = Male, 1 = Female)	0.0086	0.032	0.9749
Race (0 = White, 1 = Black)	0.0272	0.100	0.9204
HIV Status × Gender	−0.4644	1.485	0.1428
HIV Status × Race	0.2128	0.578	0.5654
Gender × Race	−0.0358	0.105	0.9168

Note: B = Unstandardized coefficient; *t* = *t*-value; *p* = *p*-value.
